# Cardiovascular biomarkers in pregnancy with diabetes and associations to glucose control

**DOI:** 10.1007/s00592-022-01916-w

**Published:** 2022-07-07

**Authors:** Daniel P. Jacobsen, Ragnhild Røysland, Heidi Strand, Kjartan Moe, Meryam Sugulle, Torbjørn Omland, Anne Cathrine Staff

**Affiliations:** 1 Division of Obstetrics and Gynaecology, Oslo University Hospital, Kirkeveien 166, PO Box 4956, 0424 Nydalen, Oslo, Norway; 2 Multidisciplinary Laboratory Medicine and Medical Biochemistry, Akershus University Hospital, Lørenskog, Norway; 3 Faculty of Medicine, Institute of Clinical Medicine, University of Oslo, Oslo, Norway; 4 Department of Obstetrics and Gynaecology, Bærum Hospital, Vestre Viken HF, Bærum, Norway; 5 Division of Medicine, Akershus University Hospital, Lørenskog, Norway

**Keywords:** Diabetes, GDF-15, Gestational diabetes, NT-proBNP, Pregnancy, Cardiac troponin T, HbA1c

## Abstract

**Aim:**

Cardiovascular disease (CVD) is a leading cause of death in both men and women. Type 1 and 2 diabetes mellitus (DM1 and DM2) are well-known risk factors for CVD. In addition, gestational diabetes mellitus (GDM) is a female sex-specific risk factor for CVD. Here, we measure circulating concentrations of cardiac troponin T (cTNT), N-terminal pro-B-type natriuretic peptide (NT-proBNP) and growth differentiation factor 15 (GDF-15) during pregnancy—a window of time often referred to as a cardiovascular stress test for women.

**Methods:**

This study utilized data from 384 pregnant women: 64 with DM1, 16 with DM2, 35 with GDM and 269 euglycemic controls. Blood was predominantly sampled within a week before delivery. Cardiovascular biomarker concentrations were measured in serum using electrochemiluminescence immunoassay.

**Result:**

Circulating cTnT levels were higher in women with DM1, DM2 and GDM as compared to controls, whereas NT-proBNP and GDF-15 levels were only increased in women with DM1. Glucose dysregulation, assessed by third trimester HbA1c levels, positively correlated with all three CVD biomarker levels, whereas pregestational body mass index correlated negatively with GDF-15.

**Conclusions:**

Our results support the presence of myocardial affection in women with diabetic disorders during pregnancy. Although pregestational DM1 in this study was associated with the most adverse CVD biomarker profile, women with GDM displayed an adverse cTnT profile similar to what we found in women with pregestational DM2. This supports that women with GDM should be offered long-term intensified cardiovascular follow-up and lifestyle advice following delivery, similarly to the well-established CV follow-up of women with pregestational DM.

**Supplementary Information:**

The online version contains supplementary material available at 10.1007/s00592-022-01916-w.

## Introduction

Cardiovascular disease (CVD) remains a leading cause of death in both men and women, but sex-specific mechanisms and risk factors have so far been underinvestigated [1]. A recent review summarizes that pathophysiologic mechanisms in diet-induced obesity and cardiometabolic disorders (i.e., heart failure, atrial fibrillation and ischemic heart disease) affect women and men’s hearts differently [2]. Diabetes mellitus (DM) is a cardiovascular disease (CVD) risk factor that affects both sexes [3], but is particularly worrisome for women [4–6]. Pregnancy represents a female sex-specific risk for developing diabetes, as women may develop gestational diabetes mellitus (GDM) during pregnancy. GDM resolves postpartum but confers a high risk for developing diabetes mellitus type 2 (DM2) later in life [7, 8]. In order to screen for DM2 development, current clinical guidelines therefore recommend a postpartum HbA1c test in women after GDM (e.g., 4 months after delivery) followed by annual HbA1c testing [9, 10]. Insulin resistance (i.e., DM2 and GDM) and CVD may be linked by several underlying factors, such as obesity, inflammation, endothelial dysfunction, hypertension and dyslipidemia. However, DM1 also confers increased cardiovascular risk [3], and DM1 and DM2 are associated with loss of cardiac innervation [11], as well as interstitial collagen deposits, resulting in cardiac wall stiffening and diastolic dysfunction [12]. In addition, heart disease generally manifests in different ways in diabetics and non-diabetics [13], further implicating a causal relationship between diabetes and cardiovascular disease.

In order to combat the high and increasing societal burden of CVD, it is necessary to develop ways of accurately identifying individuals at risk during the preclinical period. This includes developing less invasive methods to determine future or ongoing cardiac stress. We have previously shown that commonly used cardiovascular risk calculators are inadequate in assessing cardiovascular disease risk one-year postpartum following GDM [14]. In addition to blood pressure, blood lipids and exercise tests, molecular biomarkers such as circulating cardiac troponin T (cTnT) [15], N-terminal pro-B-type natriuretic peptide (NT-proBNP) [16] and growth-differentiation factor 15 (GDF-15) [17] are valuable tools in the diagnosis of preclinical heart disease. Sex-specific cut-off values for cTnT and NT-proBNP that may be used for simplified detection of preclinical cardiac disease are still lacking [2]. This despite well-known differences in levels between women and men [18] and an increased prognostic value of these markers in women [19, 20].

Soluble fms-like tyrosine kinase-1 (sFlt-1) and placental growth factor (PlGF) are predominantly expressed by the placenta during pregnancy. The antiangiogenic sFlt-1 is a decoy receptor for the proangiogenic PlGF [21]. Thus, the relative circulating levels of sFlt-1 and PlGF reflects the pregnant woman’s angiogenic profile, and we argue that a high sFlt-1/PlGF ratio is a marker of syncytiotrophoblast stress and general placental dysfunction [22].

Pregnancy has been described as a cardiovascular stress test [23], and may thus serve as an opportunity to identify women at risk for diabetes and premature cardiovascular disease. Early identification of women at risk provides opportunities for more intensified follow-up and preventive measures, at a young age where initial vascular changes (i.e., early stages of atherosclerosis) may be more reversible. Here, we measure circulating concentrations of cTnT, NT-proBNP and GDF-15 in women with DM1, DM2 or GDM during pregnancy, and compare these to healthy control pregnant women. We hypothesized that the epidemiological excessive risk of premature CVD among women with diabetes—especially DM1—will be reflected by CVD risk biomarkers during pregnancy.

## Methods

### Study subjects

As previously described [24], women who had not yet gone into active labor were recruited to the Oslo Pregnancy biobank (OPB) [25], either upon admission for cesarean section or as outpatients followed up for pregnancy complications during the second half of pregnancy. Recruitment in pregnancy was mainly restricted by availability of study personnel, as almost none of the women approached declined participation. We included 384 women from the OPB prior to delivery: 64 with DM1, 16 with DM2, 35 with GDM and 269 euglycemic women (controls). Only women with singleton pregnancies, and no history of hypertension or other inflammatory diseases (e.g., autoimmunity or cancer) were included. Diabetes mellitus (DM) was defined according to the World Health Organization criteria at the time of inclusion [26], and diagnoses were retrieved from the individual medical charts. The patients with pregestational diabetes attended antenatal follow-up at the Oslo University Hospital according to routine, with assessment by endocrinologist, obstetrician and midwife.

The in-patient hospital blood pressure (BP) was based on repeated measurements with a validated device for pregnancy (Dinamap Pro, 100VE, GE Medical Systems Information Technology, Inc. Milwaukee, Wisconsin, USA), as previously described [14]. Offspring sex and gestational age specific birth weight percentiles were calculated according to Norwegian ultrasound-based percentiles [27].

### Biomarker measurement

All maternal pregnancy blood samples were drawn predominantly within a week prior to delivery. Median gestational age at sampling was 37 + 0 for women with DM1, 37 + 1 for women with DM2, 38 + 5 for women with GDM and 39 + 0 for euglycemic controls (Table 1). Serum blood samples were thawed and analyzed for levels of cTnT, NT-proBNP and GDF-15 at the department for Multidisciplinary Laboratory Medicine and Medical Biochemistry at Akershus University Hospital, using electrochemiluminescence immunoassay Elecsys on the **cobas e** 801 platform (Roche Diagnostics, Rotkreuz, Switzerland). For cTnT, NT-proBNP and GDF-15 measuring ranges were 3–100,000 ng/L, 5–35,000 ng/L and 400–20,000 ng/L, respectively. Samples with GDF-15 levels > 20,000 ng/L were diluted 1:20 and reanalyzed. Biomarker values above or below measuring ranges were set to maximum or minimum possible measuring value, respectively. HbA1c vas available from the third trimester in 62 women with DM1, 15 with DM2, 33 with GDM and 3 controls.

**Table 1:** Pregnancy cohort (*n* = 115): clinical pregnancy characteristics and biomarker levels, by study groups

	Control, *n* = 269	DM1, *n* = 64	DM2, *n* = 16	GDM, *n* = 35
Age at inclusion (years)	33.8 (30.7–36.4)	32.4 (28.5–36.1)*	34.8 (29.2–37.6)	35.2 (31.9–39.5)
BMI before pregnancy (kg/m^2^)	22.4 (20.6–25.3)	24.2 (22.0–27.3)**	28.2 (25.2–29.6)***	25.4 (22.8–29.8)***
Obesity before pregnancy (BMI ≥ 30 kg/m^2^)	19 (7%)	8 (12%)	3 (19%)	8 (23%)**
BMI at inclusion (kg/m^2^)	27.7 (25.3–31.2)	29.1 (27.0–33.6)**	33.6 (29.9–37.1)***	30.5 (27.4–34.3)**
Gestational age at inclusion (weeks + days)	39 + 0 (38 + 5–39 + 2)	37 + 0 (36 + 1–38 + 1)***	37 + 1 (36 + 2–38 + 1)***	38 + 5 (37 + 4–39 + 0)***
Gestational age at delivery (weeks + days)	39 + 0 (38 + 5–39 + 2)	38 + 2 (36 + 4–39 + 0)***	38 + 4 (37 + 1–39 + 3)	38 + 6 (38 + 2–39 + 1)
Neonatal weight (grams)	3474 (3195–3731)	3835 (3300–4189)***	3984 (3314–4442)	3802 (3400–4260)**
Neonatal weight (percentile)	62.4 (34.2–82.2)	92.5 (67.2–99.6)***	93.8 (31.5–99.5)	86.1 (50.8–99.1)**
Newborn sex (girl/boy)	122/147	17/47**	4/12	15/20
Primiparous	107 (40%)	30 (47%)	8 (50%)	13 (37%)
Systolic BP < week 20 (mmHg)	110 (102–117)	115 (109–120)***	122 (113–124)***	115 (105–123)*
Diastolic BP < week 20 (mmHg)	68 (62–73)	70 (65–73)	71 (67–79)*	70 (64–75)
Systolic BP at inclusion (mmHg)	120 (114–131)	135 (117–148)***	137 (122–154)***	120 (110–130)
Diastolic BP at inclusion (mmHg)	75 (69–82)	80 (70–88)**	88 (80–96)***	71 (67–80)
Hypertension at inclusion^a^	32 (12%)	30 (47%)***	8 (50%)***	4 (11%)
3. Trimester HbA1c (%)	5.2	6.4 (5.9–6.8)	6.3 (6.1–6.7)	5.8 (5.4–6.2)
3. Trimester HbA1c (mmol/mol)^b^	33 (NA)	46 (41–51)	45 (43–50)	40 (36–44)
Hba1c ≥ 6% (42 mmol/mol)^b^	0/3	46/62	13/15	11/33
sFlt-1 (pg/mL)	3676 (2747–5168)	6202 (3830–7860)***	5393 (3258–8907)***	4220 (2964–5391)
PlGF (pg/mL)	171 (110–297)	136 (79–183)***	128 (95–362)	210 (141–476)
sFlt-1/PlGF	22 (10–42)	53 (18–88)***	29 (13–89)	22 (7–37)
cTnT (ng/L)	3 (3–4)	6 (4–8)***	5 (3–8)***	4 (3–5)*
NT-proBNP (ng/L)	29 (19–42)	57 (34–124)***	36 (17–93)	26 (14–52)
GDF-15 (ng/L)	88,344 (66,960–117,685)	111,871 (94,601–147,348)***	99,966 (62,653–114,599)	103,771 (70,873–123,354)

Values are given as medians (and interquartile ranges) or numbers (and percentages). Each subgroup was compared to controls using the Mann–Whitney *U* test (continuous variables) and the Fisher’s exact test (categorical variables), *p < 0.050, **p < 0.010, ***p < 0.001. At “inclusion” signifies at the time for blood sampling used in the analyses

*DM1* Diabetes mellitus type 1, *DM2* Diabetes mellitus type 2, *GDM* Gestational diabetes mellitus, *BMI* Body mass index, *BP* Blood pressure, *cTnT* Cardiac troponin T, *NT-proBNP* N-terminal fragment of the B-type natriuretic peptide prohormone, *GDF-15* Growth differentiation factor 15

^a^Hypertension: Blood pressure ≥ 140 mmHg systolic and/or ≥ 90 mmHg diastolic

^b^No tests of significance due to missing data: HbA1c measurements were available for 3/269 controls, 62/64 with DM1, 15/16 with DM2 and 33/35 with GDM

The maternal PIGF and sFlt-1 serum concentrations from predelivery blood samples were quantified at the Department of Medical Biochemistry, Oslo University Hospital, on a **cobas e** 801 (Roche Diagnostics, Rotkreuz, Switzerland), using the fully automated Elecsys PlGF and sFlt-1 system, according to the manufacturer´s instructions.^1^ Alternatively, the PlGF and sFlt-1 proteins were measured by the same Roche reagents, using an Elecsys 2010 Modular Analytics E170 or a **cobas e** 601 (Roche Diagnostics, Rotkreuz, Switzerland). All concentrations were within the measuring ranges of the PlGF and sFlt-1 assays (3–10,000 pg/mL and 10–85,000 pg/mL, respectively). The coefficients of variation were ≤ 2.1% for PlGF and ≤ 1.8% for sFlt-1.

### Statistical analysis

Continuous variables are presented as medians and interquartile ranges and categorical variables as counts (percent). Comparisons between groups were conducted using the nonparametric Mann–Whitney *U* test for continuous variables and the Fisher’s exact test for categorical variables. Spearman’s rank correlation was used to assess the relationships between HbA1c and cardiovascular biomarkers. Statistical analyses were performed using SPSS version 22.0 (IBM). The level of significance was set at *p* < 0.05.

## Results

### Pregnancy phenotypes and placenta-cardiovascular biomarkers

Descriptive statistics as well as biomarker levels during pregnancy are shown in Table 1. The groups of women with DM1, DM2 and GDM had higher prepregnancy BMI as well as higher BMI at delivery, when compared to controls. The group with GDM also had a higher proportion of obese women (BMI ≥ 30 kg/m^2^). Median birthweight percentiles were higher in the DM1 and GDM groups as compared to controls. At delivery, women with DM1 or DM2 had higher median systolic and diastolic blood pressures as compared to controls, as well as significantly higher prevalences of hypertension (systolic blood pressure ≥ 140 mmHg and/or diastolic blood pressure ≥ 90 mmHg).

When compared to controls, cTnT was higher in DM1, DM2 and GDM, while NT-proBNP and GDF-15 were only significantly higher in DM1. Women with DM1 also had significantly higher levels of cTnT (*p* < 0.001) and NT-proBNP (*p* < 0.001) than women with GDM.

As expected, women with hypertension at inclusion (systolic blood pressure ≥ 140 mmHg and/or diastolic blood pressure ≥ 90 mmHg) had significantly higher levels of cTnT (*p* < 0.001), NT-proBNP (*p* < 0.001) and GDF-15 (*p* = 0.012, Supplemental Fig. 1). Elevated cTnT and NT-proBNP, but not GDF-15, levels were associated with hypertension when women with diabetes and controls were analyzed separately as well (results not shown).

Among women with diabetes, BMI before pregnancy was negatively correlated with NT-proBNP (*r*_s_ = − 0.261, *p* = 0.005) and GDF-15 (*r*_s_ = − 0.241, *p* = 0.010). Among controls, BMI before pregnancy was only negatively correlated with GDF-15 (*r*_s_ = − 0.167, *p* = 0.006). Moreover, pregestational obesity (BMI ≥ 30 kg/m^2^ before pregnancy) was associated with significantly lower levels of GDF-15 among diabetics (*p* = 0.009, Supplemental Fig. 2) and controls (*p* = 0.006, Supplemental Fig. 3).

**Fig. 1:**
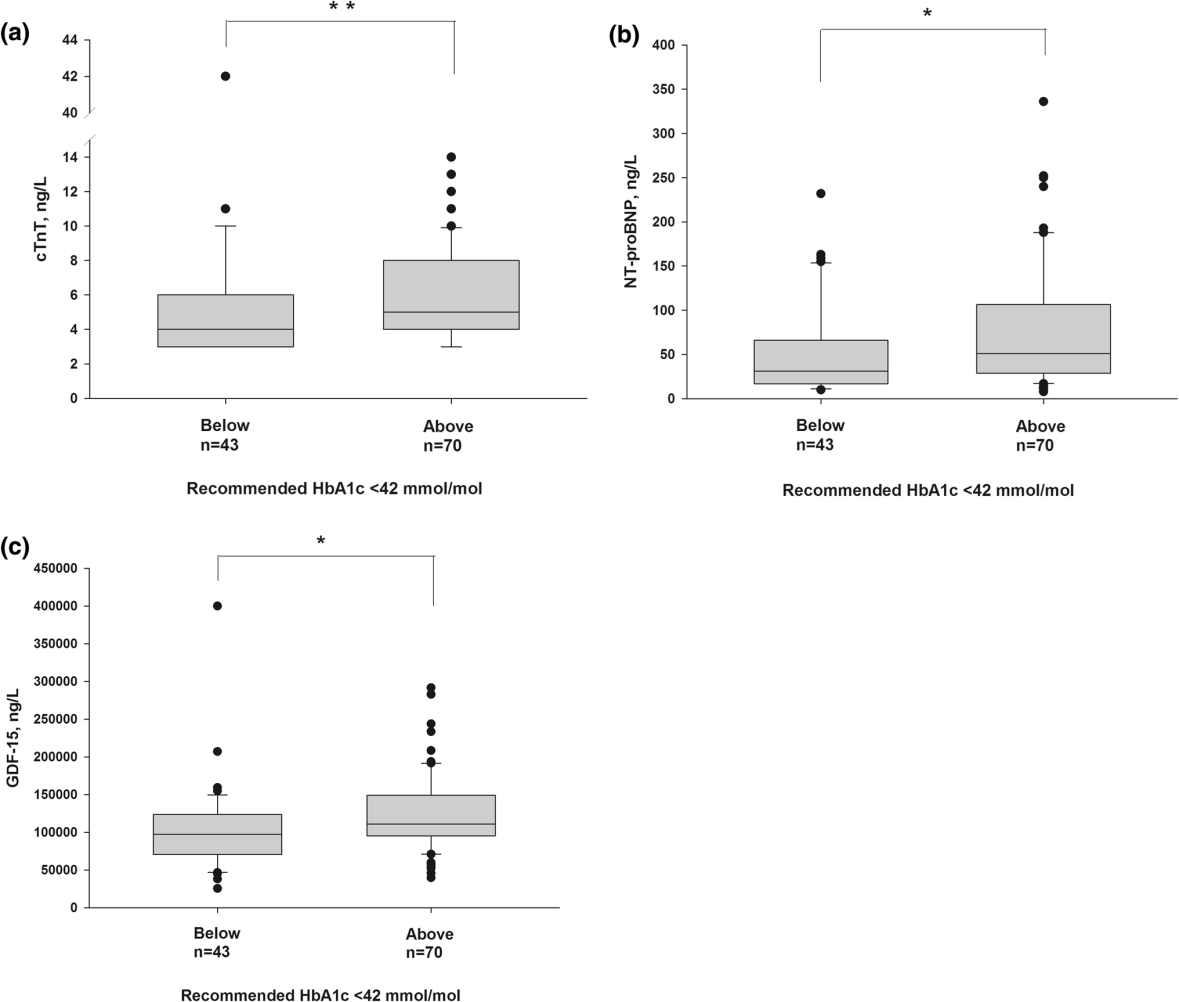
Boxplots of circulating **A** cardiac troponin T (cTnT), **B** N-terminal pro-Brain Natriuretic Peptide (NT-proBNP) and **C** growth differentiation factor 15 (GDF-15) levels, all in ng/L. Pregnant women with available third trimester HbA1c measurements were categorized into groups based on Norwegian guidelines for glucose control during pregnancy: HbA1c < 42 mmol/mol versus ≥ 42 mmol/mol. Biomarker concentrations are shown in boxplots as 10th percentile (lower whisker), 25th percentile, median (horizontal box line), 75th percentile, 90th percentile (upper whisker), as well as outliers. Groups were compared using the Mann–Whitney *U* test, *p < 0.050, **p < 0.010

Norwegian guidelines for glucose control during pregnancy recommend a third trimester HbA1c level below 6% (42 mmol/mol) for women with pregestational diabetes [28]. In our cohort, most of the women had HbA1c levels above this goal; 46 of 62 with DM1, 13 of 15 with DM2 and 11 of 33 with GDM. The 3 controls with available HbA1c measurements had as expected values below 6% (42 mmol/mol). In the total cohort, HbA1c correlated positively with cTnT (*r*_s_ = 0.284, *p* = 0.002), NT-proBNP (*r*_s_ = 0.214, *p* = 0.023) and GDF-15 (*r*_s_ = 0.271, *p* = 0.004). Moreover, women with HbA1c values at or above the recommended 6% (42 mmol/mol) had significantly higher levels of cTnT (*p* = 0.008), NT-proBNP (*p* = 0.019) and GDF-15 (*p* = 0.013) as compared to women below this threshold (Fig. 1).

Median sFlt-1 was increased in both DM1 and DM2, but only women with DM1 had decreased median PlGF and a significantly dysregulated sFlt-1/PlGF ratio relative to controls (Table 1). We recently demonstrated a correlation between sFlt-1/PlGF and the CVD markers cTnT and NT-proBNP in women with hypertensive disorders during pregnancy and healthy controls (article in review). Among diabetics (DM1, DM2 and GDM), we again observe a correlation between sFlt-1/PlGF and cTnT (*r*_s_ = 0.459, *p* = 0.000) and between sFlt-1/PlGF and NT-proBNP (*r*_s_ = 0.504, *p* = 0.000). The association between sFlt-1/PlGF and GDF-15 was not significant (*r*_s_ = 0.114, *p* = 0.225).

## Discussion

This study shows that DM1 is associated with the highest levels of markers of cardiovascular injury and dysfunction during pregnancy, among diabetic disorders. This is in line with previous observations of increased cardiovascular disease risk and all-cause mortality in people with DM1 as compared to both non-diabetics and people with DM2 [29]. Our results also add to previous reports of elevated cardiovascular biomarkers in women with DM2 and GDM during pregnancy, and underscore the importance of glucose control during pregnancy.

Elevated circulating levels of cardiac troponins in women with DM1, DM2 and GDM, indicating myocardial injury, have been well documented previously [30, 31]. Even prediabetic patients may have higher levels of cTnT in the circulation [32]. Moreover, in both men and women with DM2, cTnT is correlated with advanced glycation end-products (AGE), markers of oxidative stress, and arterial pulse wave reflection [33], and may serve as a biomarker for increased risk of coronary artery disease in patients with diabetes [34, 35]. Our previous report of elevated circulating AGE in pregnancies affected by diabetes [36] combined with our present findings of elevated cTnT in the same groups underscore the presence of cardiovascular dysfunction in diabetes, also during pregnancy. This adds further support to the Obstetrics Guidelines 2020 of the Norwegian Society of Gynecology and Obstetrics (NGF), which recommend supplementing the traditional 4 month postpartum (and later annual) HbA1c testing for DM2 development with a general assessment of cardiovascular health [10]. The NGF guideline suggests similar cardiovascular follow-up by a general practitioner in line with what the Society also suggests after a pregnancy complicated by preeclampsia [37]. The clinical impression is that these recommendations are not followed up, possibly due to limited knowledge of the long-term effects of GDM on other risk factors for cardiovascular disease than glucose control.

Outside of pregnancy, DM1 [38, 39] and DM2 [40] are associated with elevated NT-proBNP levels. Moreover, NT-proBNP may act as an independent risk factor for CVD in patients with DM1 [41] and DM2 [42]. This upregulation may be due to the structural and functional cardiac changes associated with diabetes mellitus mentioned above. Here, we report elevated maternal levels of NT-proBNP in pregnancies with DM1, but no difference in NT-proBNP levels between pregnant women with DM2 and healthy controls. This may be due to the low number (16) of women with DM2 in our study. The regulation of NT-proBNP in GDM is a disputed topic. While Mert and colleagues show clear increases in NT-proBNP levels in GDM [31], two other studies failed to show the same association [43, 44]. Our findings are in line with these latter reports, suggesting that NT-proBNP levels in women with GDM do not reflect the epidemiologically increased risk for future CVD in this group [45].

Unlike cTnT and NT-proBNP, GDF-15 is highly expressed in the placenta [46]. In fact, maternal circulating GDF-15 correlates with placental GDF-15 mRNA levels [25]. In a partly overlapping cohort (84 subjects in common: 27 DM1, 8 DM2, 12 GDM and 37 controls), we have previously shown increased levels of GDF-15 during pregnancy in diabetic pregnancies collectively [25]. In addition, other studies have shown elevated GDF-15 levels in DM2 [47] and GDM separately [48]. GDF-15 is also elevated before onset of DM2, but is not an independent predictor of the disease [49]. Rather, researchers argue that confounding factors underlie elevated GDF-15 levels as well as increased risk of DM2. In the present study, only the group of women with DM1 had significantly higher median level of GDF-15 as compared to the control group. Although the levels of circulating GDF-15 were elevated in women with DM2 and GDM as well, these were not significantly different from controls. This may be partly due to the low number of participants in these groups.

GDF-15 suppresses appetite in animal models [50, 51] and is inversely correlated with BMI in humans outside pregnancy [52]. In pregnancy, a state during which GDF-15 levels increase 200-fold, there is still a negative correlation between GDF-15 and BMI [53]. Here, we report a negative association between GDF-15 prior to delivery and pregestational BMI. In addition, we report no correlation between pregestational obesity and cTnT or NT-proBNP, suggesting that the observed associations between elevated CVD risk markers and diabetes are not confounded by BMI.

Hyperglycemia is known to cause substantial damage to the glycocalyx [54]. Destruction of this protective proteoglycan layer may promote atherosclerosis in larger arteries [55], as well as microvascular disease in arterioles and capillaries [56]. In addition, hyperglycemia reduces NO synthesis [57], reduced endothelial progenitor cell numbers and function [58] and causes hypercoagulability [59]. Accordingly, we here demonstrate an association between the severity of glucose mismanagement during pregnancy and circulating CVD risk markers. This is in line with previous studies also showing a positive correlation between HbA1c levels and circulating troponins, NT-proBNP and GDF-15 [60–62]. These observations underscore the importance of glucose control during pregnancy and support strict HbA1c guidelines and follow-up of pregnant women with diabetes. In the present study cohort, the majority of women with DM1 and DM2, as well as a third of women with GDM presented with third trimester HbA1c measurements exceeding the Norwegian guidelines for glucose control during pregnancy for pregestational diabetes mellitus. Norway follows strict guidelines for antenatal follow-up of women with pregestational diabetes [28] and GDM [10]. These guidelines are in line with international guidelines, such as the UK NICE guidelines [9].

In line with our recent report from hypertensive pregnancies (article in review), we observe correlations across all diabetic groups in pregnancy between the sFlt-1/PlGF ratio and cTnT and NT-proBNP, adding support to our concept of crosstalk between placental function and cardiovascular health. As expected, women with DM1 had a median birthweight percentile that was significantly higher than healthy control pregnancies, which is known to associate with larger placentas. The group also displayed a significantly elevated antiangiogenic sFlt-1/PlGF ratio compared to controls. These two findings support our concept of excessive placental growth leading to microvillus overcrowding and placental syncytiotrophoblast stress, which we have postulated underlies the development of placental dysfunction and thus late-onset preeclampsia [63]. The increased risk of preeclampsia development in women with pregestational diabetes is well documented [64], and in accordance with the particularly elevated CVD risk biomarker profile in women with DM1 in the present study.

The cross-sectional nature of our study design and the lack of hard endpoints limit the interpretation of biomarker profiles to assumed CVD risk. Longitudinal studies are required to further elucidate the relationship between diabetes during pregnancy and CVD. Furthermore, many women with GDM do not develop glucose intolerance postpartum [65]. Follow-up characterization and circulating biomarker measurements in women with previous GDM is of great importance, but beyond the scope of the present study. Still, our well-characterized study cohort and the high general health status of the Norwegian population provide unique insights into the association between metabolic dysfunction, placental function and CVD biomarker levels during pregnancy.

Our results indicate the presence of myocardial injury and stress in women with diabetic disorders during pregnancy. Furthermore, we show that women with DM1 are the most affected by the cardiovascular burden of pregnancy. Still, our results suggest that women with GDM should receive targeted cardiovascular follow-up postpartum and receive lifestyle advice, not limited to annual measurement of HbA1c.

## Supplementary Information

Below is the link to the electronic supplementary material.

Supplementary file1 (TIF 12252 KB)

Supplementary file2 (TIF 12252 KB)

Supplementary file3 (TIF 12252 KB)

Supplementary file4 (TIF 12252 KB)

Supplementary file5 (TIF 12252 KB)

Supplementary file6 (TIF 12252 KB)

Supplementary file7 (TIF 12252 KB)

Supplementary file8 (TIF 12252 KB)

Supplementary file9 (TIF 12252 KB)

## Footnotes

1 *COBAS E and ELECSYS are trademarks of Roche.*
