# Supplementary figures and images for: Cardiovascular biomarkers in pregnancy with diabetes and associations to glucose control
Source: Acta Diabetol. 2022 Jul 7;59(9):1229–36. doi: 10.1007/s00592-022-01916-w (PMC9329411; doi:10.1007/s00592-022-01916-w)

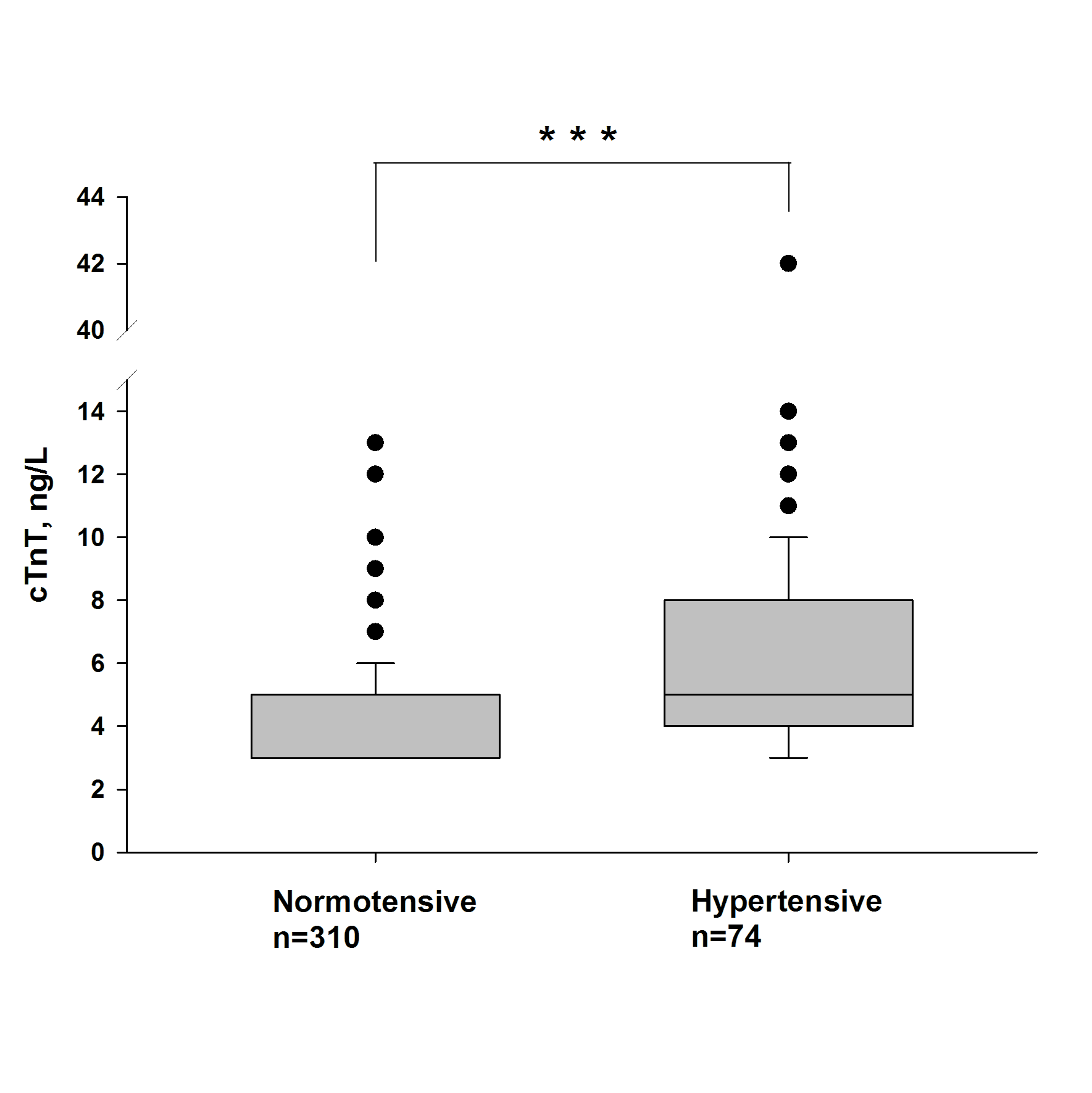

Supplement: Supplementary file 1 — Supplementary file1 (TIF 12252 KB) [file 592_2022_1916_MOESM1_ESM.tif]

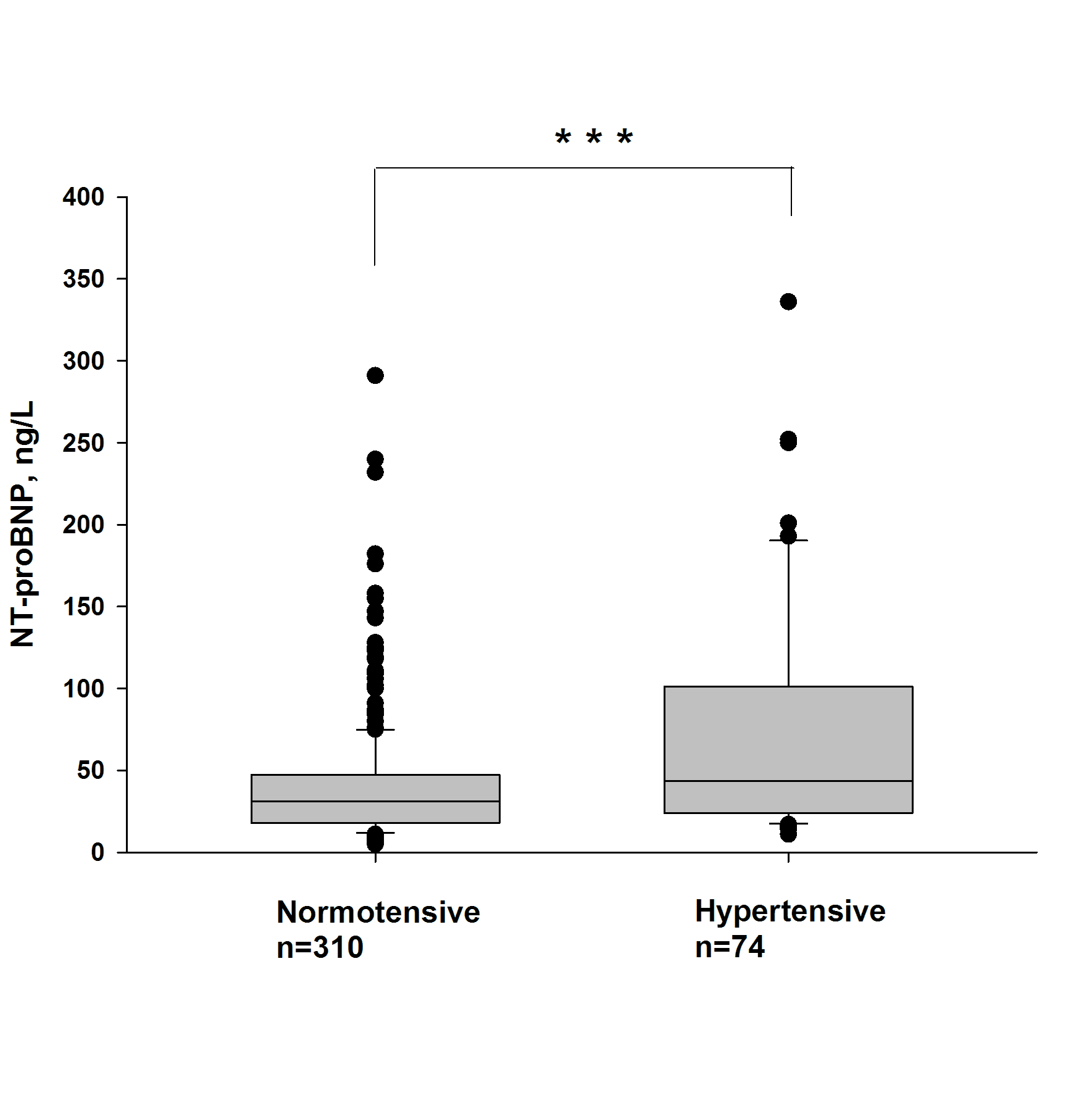

Supplement: Supplementary file 2 — Supplementary file2 (TIF 12252 KB) [file 592_2022_1916_MOESM2_ESM.tif]

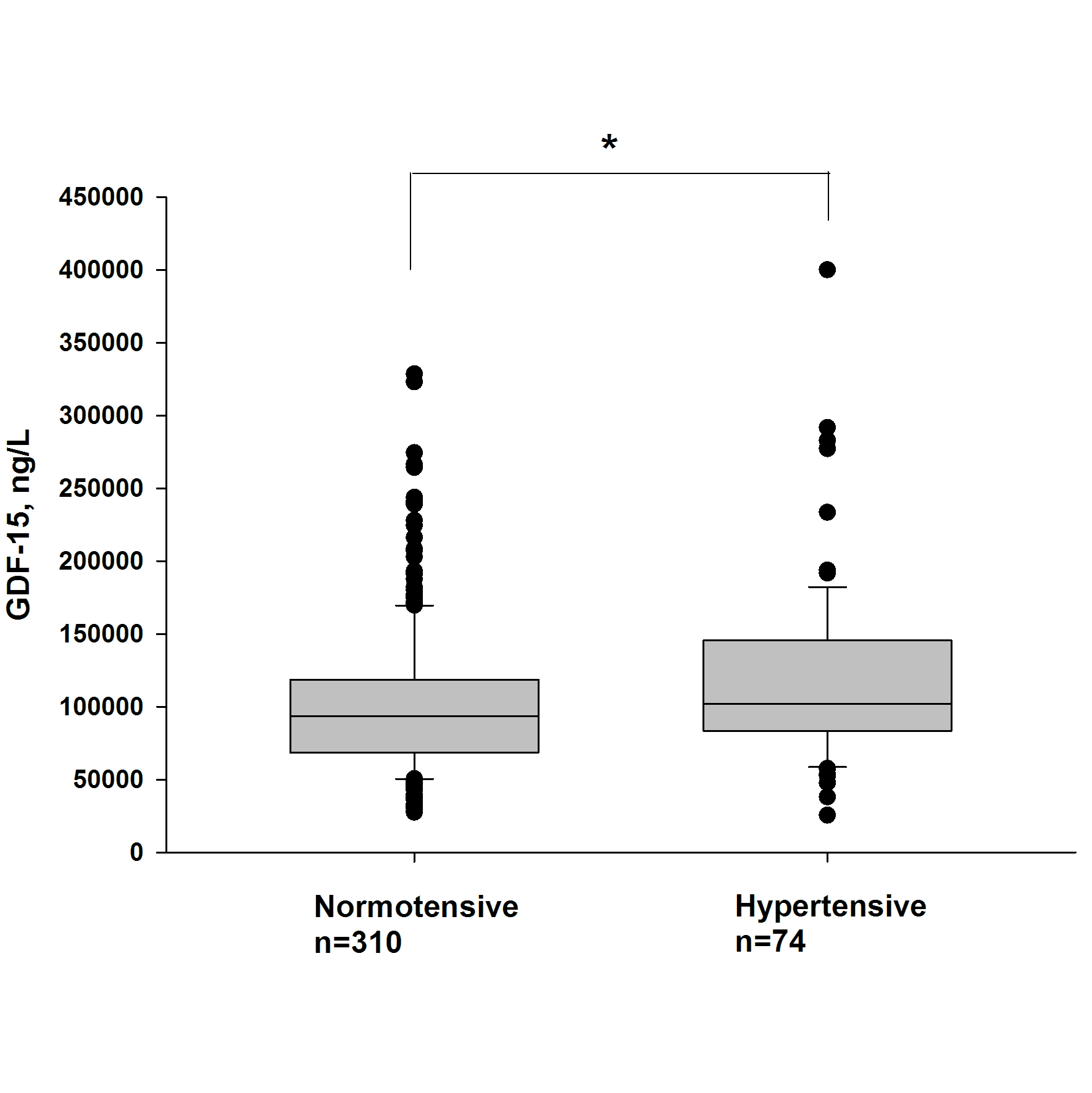

Supplement: Supplementary file 3 — Supplementary file3 (TIF 12252 KB) [file 592_2022_1916_MOESM3_ESM.tif]

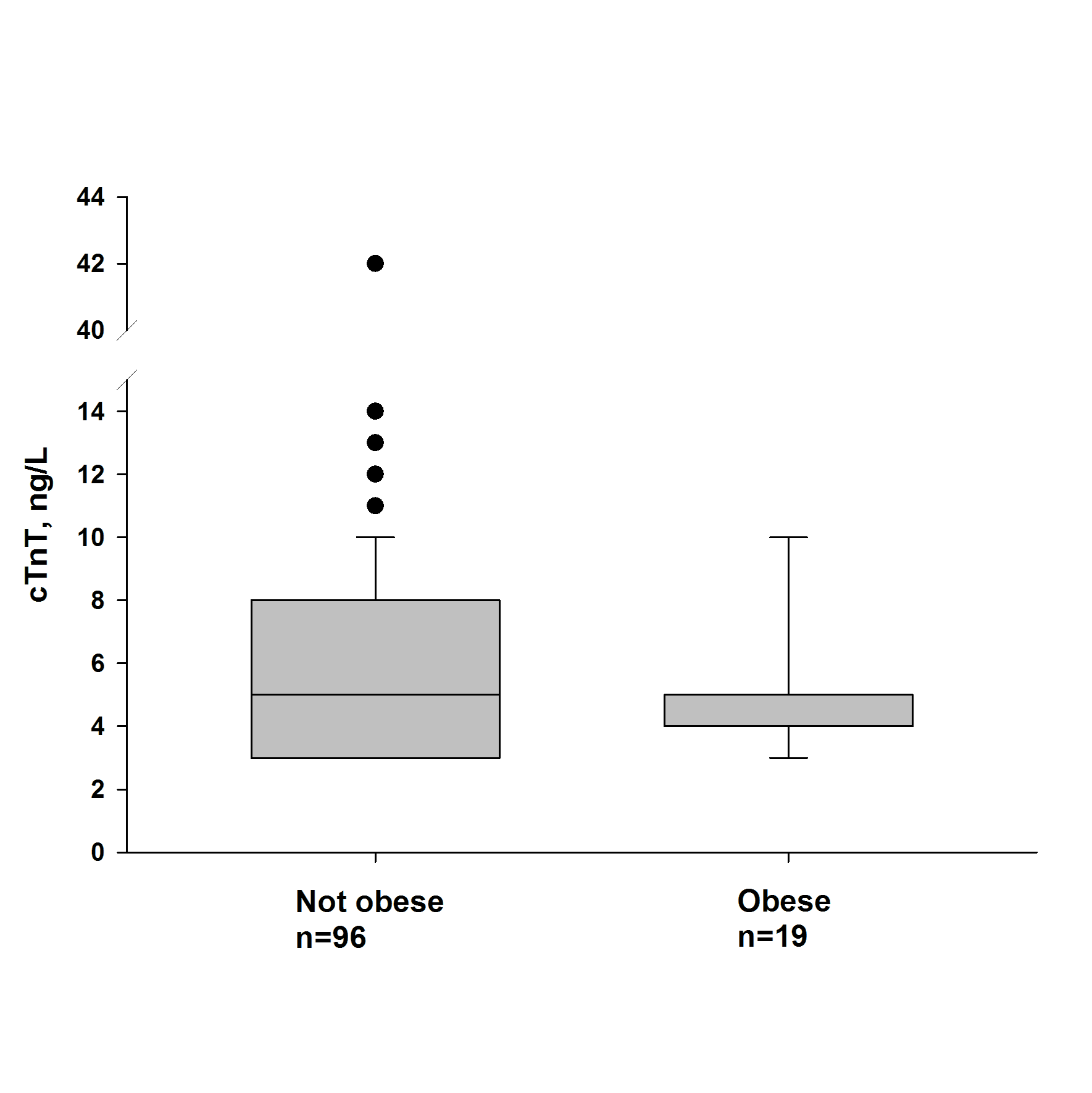

Supplement: Supplementary file 4 — Supplementary file4 (TIF 12252 KB) [file 592_2022_1916_MOESM4_ESM.tif]

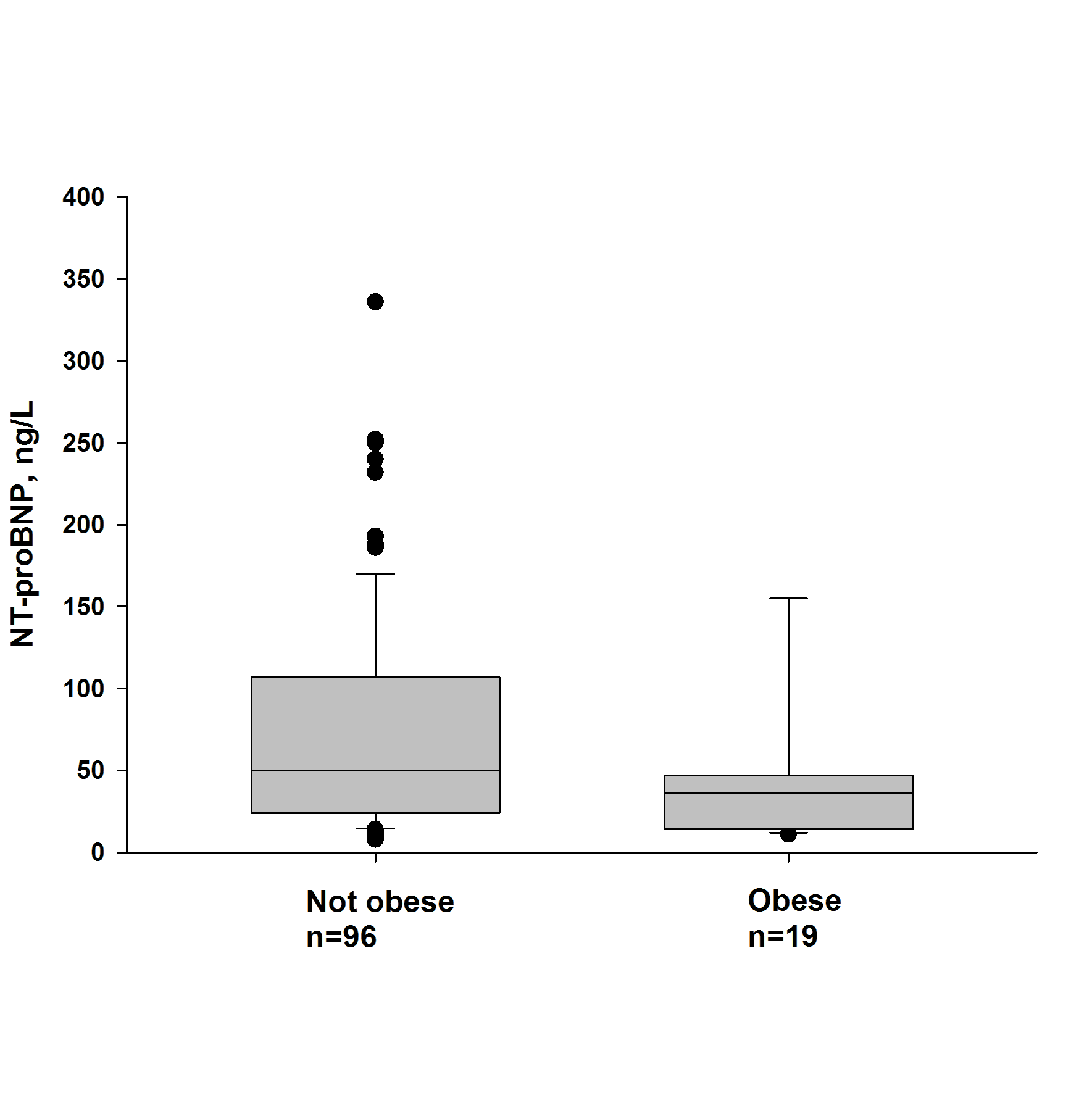

Supplement: Supplementary file 5 — Supplementary file5 (TIF 12252 KB) [file 592_2022_1916_MOESM5_ESM.tif]

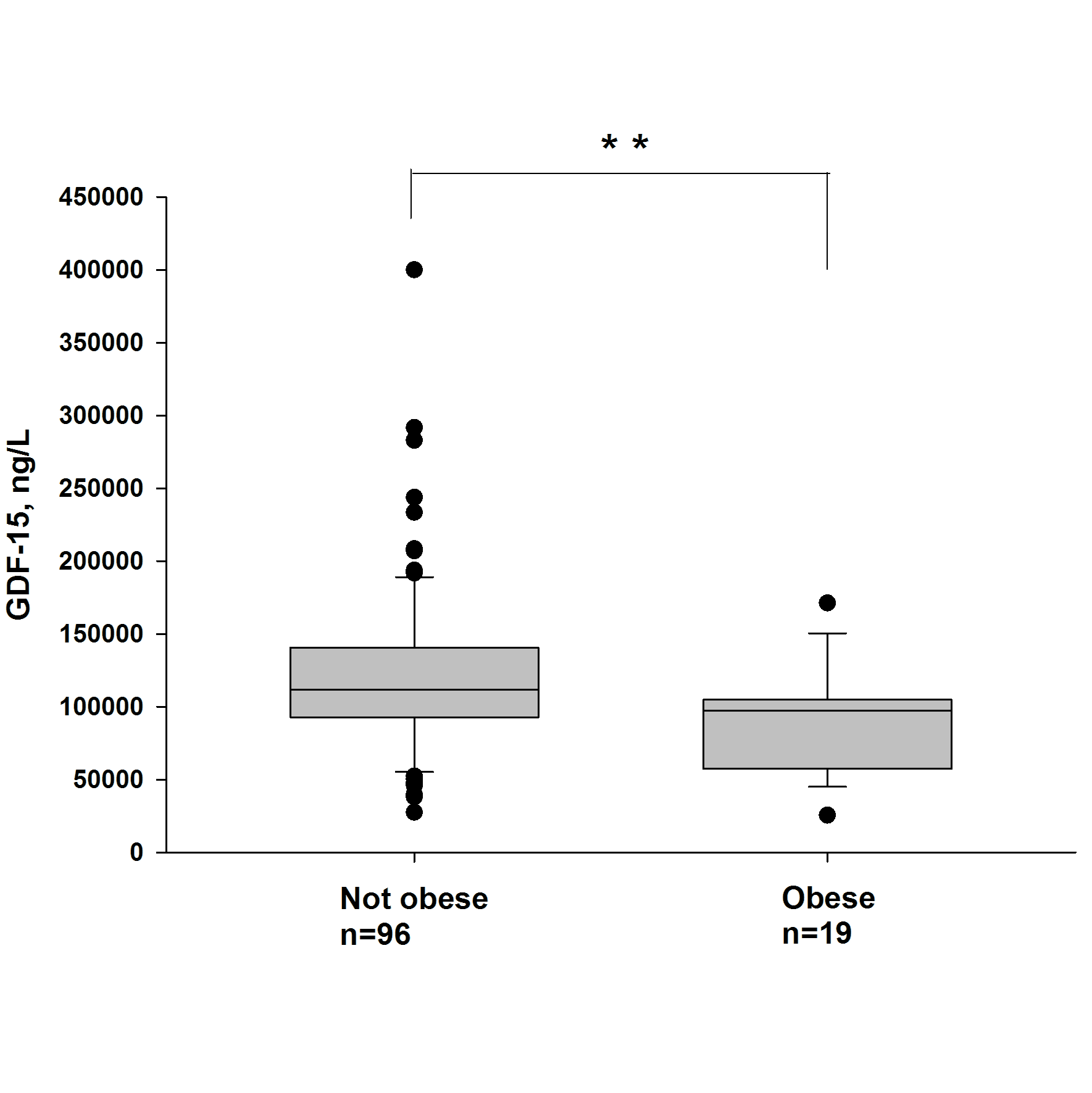

Supplement: Supplementary file 6 — Supplementary file6 (TIF 12252 KB) [file 592_2022_1916_MOESM6_ESM.tif]

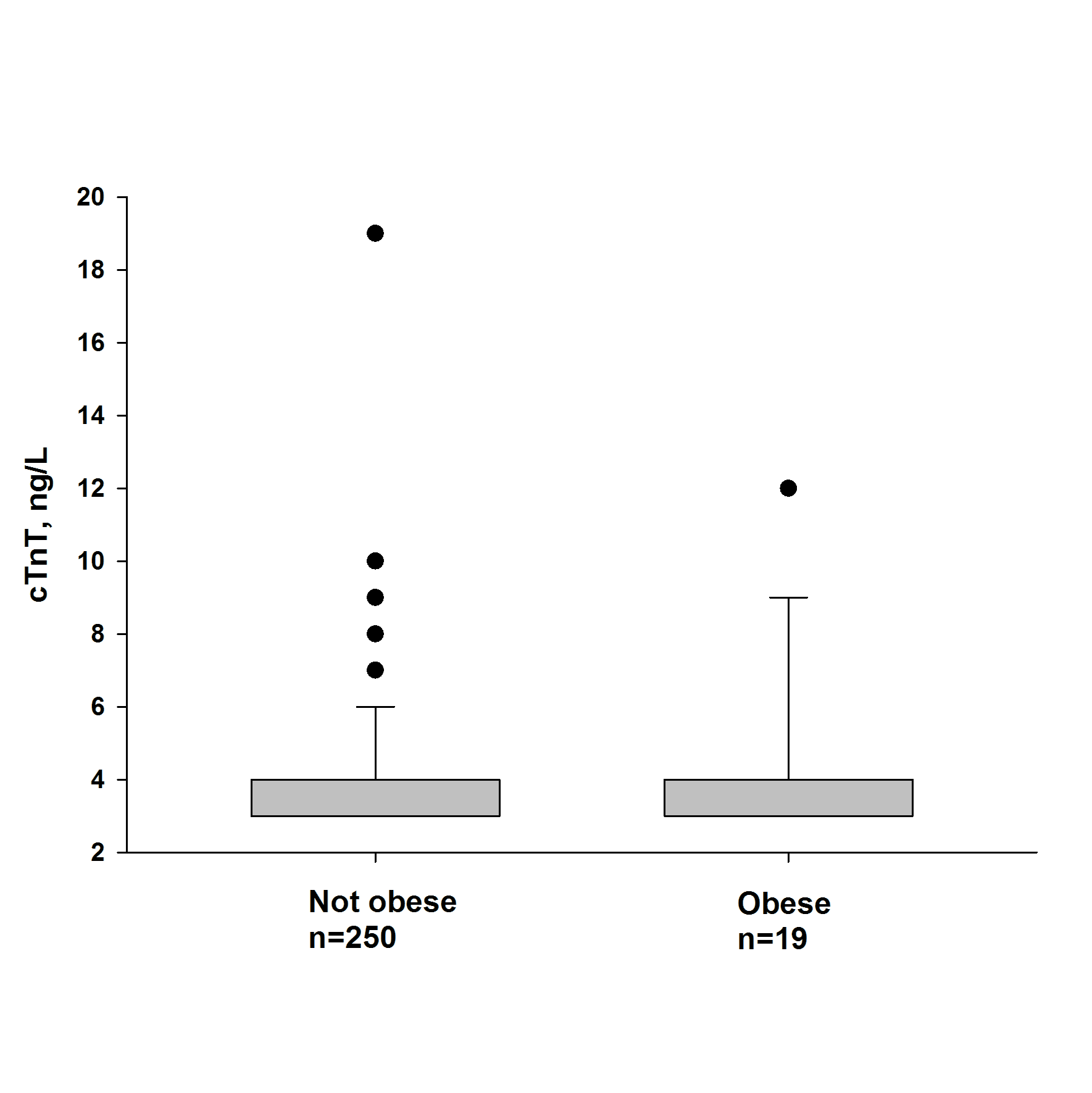

Supplement: Supplementary file 7 — Supplementary file7 (TIF 12252 KB) [file 592_2022_1916_MOESM7_ESM.tif]

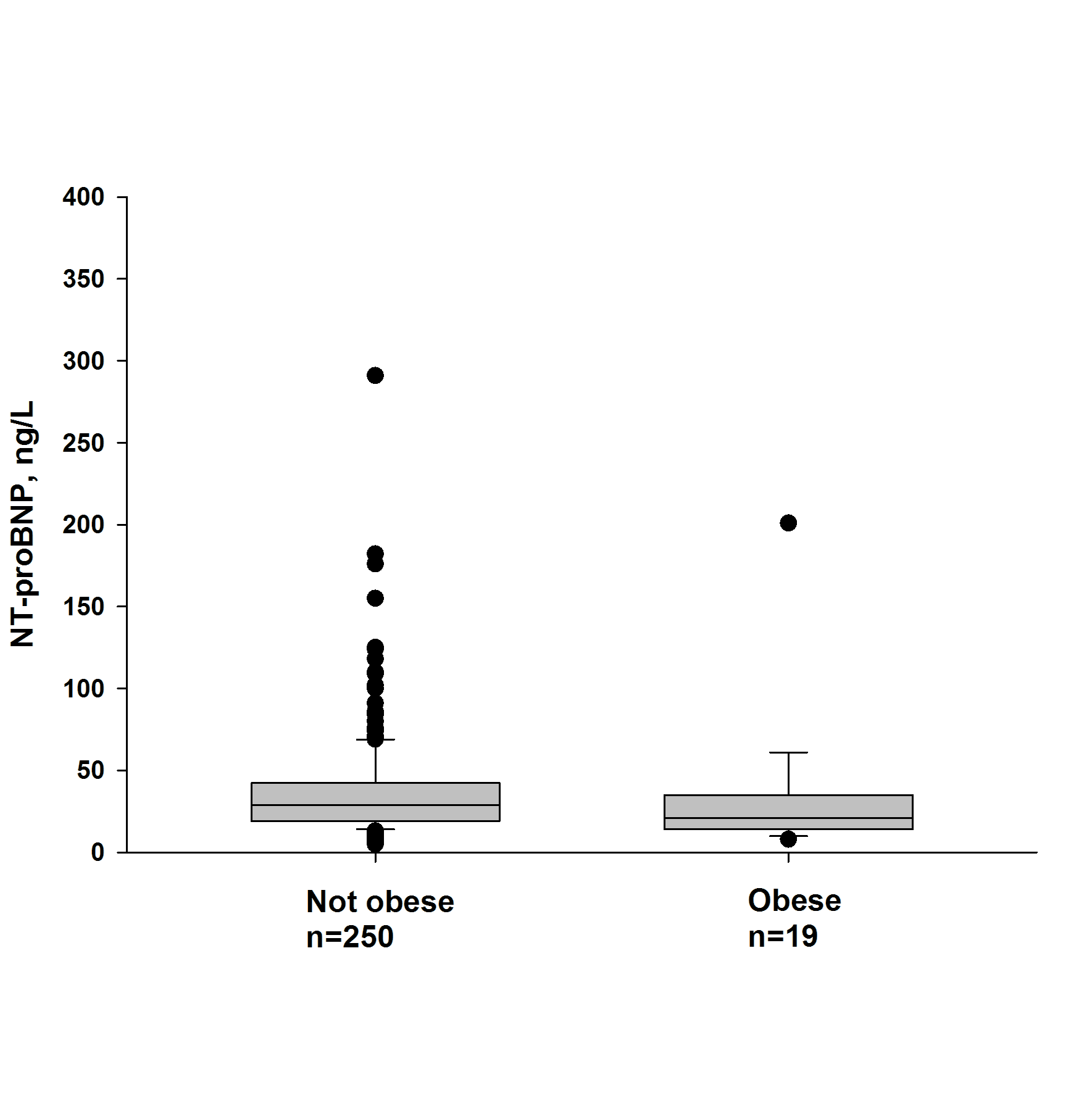

Supplement: Supplementary file 8 — Supplementary file8 (TIF 12252 KB) [file 592_2022_1916_MOESM8_ESM.tif]

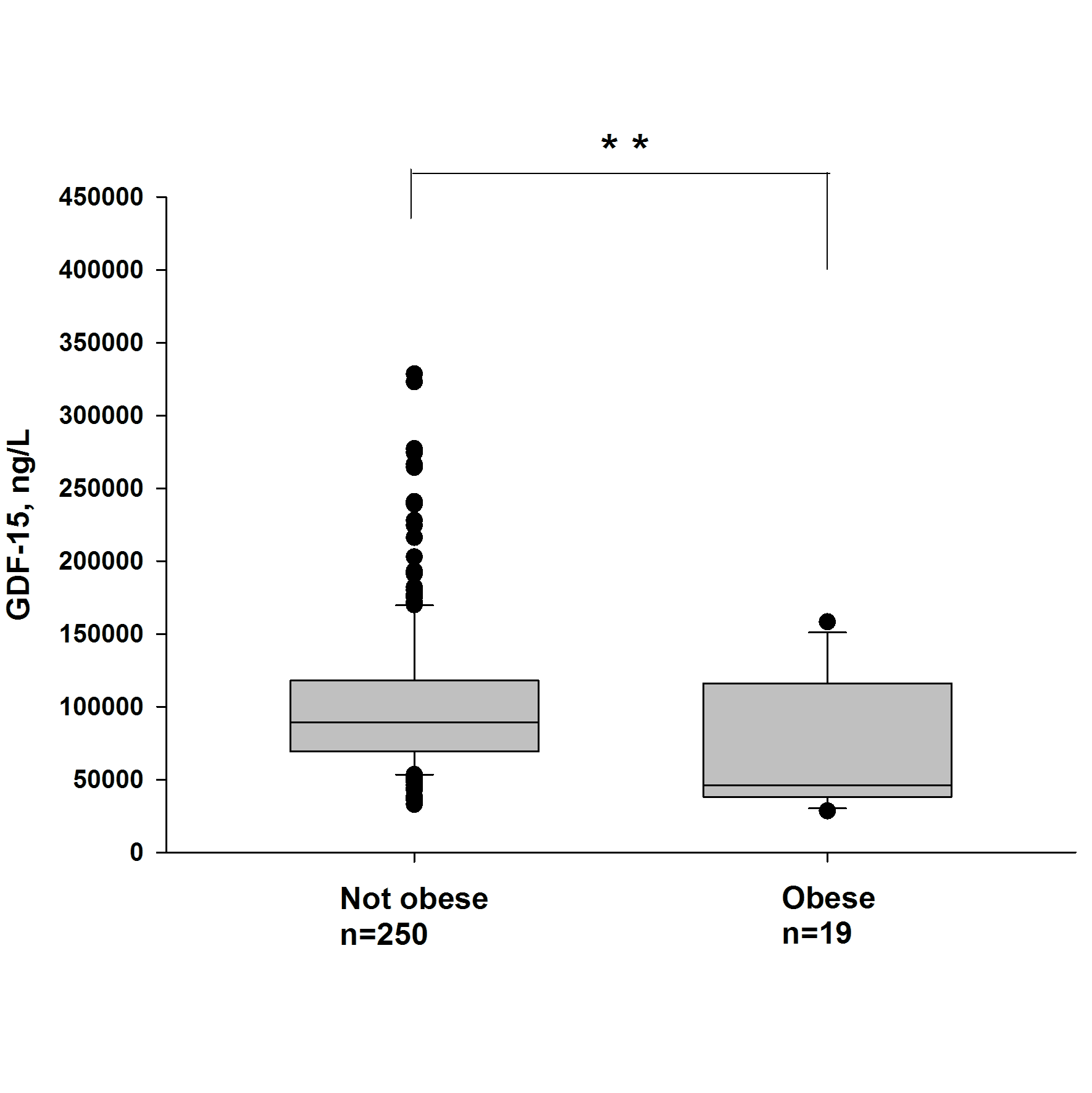

Supplement: Supplementary file 9 — Supplementary file9 (TIF 12252 KB) [file 592_2022_1916_MOESM9_ESM.tif]
